# Use of Pd-Ag Membrane Reactors for Low-Temperature Dry Reforming of Biogas—A Simulation Study

**DOI:** 10.3390/membranes13070630

**Published:** 2023-06-29

**Authors:** Matilde Albano, Luís M. Madeira, Carlos V. Miguel

**Affiliations:** 1 Department of Chemical Engineering, Faculty of Engineering, University of Porto, Rua Dr. Roberto Frias, 4200-465 Porto, Portugal; 2 Fraunhofer Portugal AWAM—Research Center for Smart Agriculture and Water Management, Régia Douro Park—Parque de Ciência e Tecnologia, 5000-033 Vila Real, Portugal; 3 LEPABE—Laboratory for Process Engineering, Environment, Biotechnology and Energy, Faculty of Engineering, University of Porto, Rua Dr. Roberto Frias, 4200-465 Porto, Portugal; mmadeira@fe.up.pt; 4 ALiCE—Associate Laboratory in Chemical Engineering, Faculty of Engineering, University of Porto, Rua Dr. Roberto Frias, 4200-465 Porto, Portugal

**Keywords:** membrane reactor, biogas, hydrogen production, dry reforming, syngas

## Abstract

Biogas is a valuable renewable energy source that can help mitigate greenhouse emissions. The dry reforming of methane (DRM) offers an alternative hydrogen production route with the advantage of using two main greenhouse gases, CO_2_ and CH_4_. However, its real application is limited mainly due to catalyst deactivation by coke formation and the reverse water gas shift (RWGS) reaction that can occur in parallel. Additionally, the typical dry reforming temperature range is 700–950 °C, often leading to catalyst sintering. A low-temperature DRM process could be in principle achieved using a membrane reactor (MR) to shift the dry reforming equilibrium forward and inhibit the RWGS reaction. In this work, biogas reforming was investigated through the simulation of MRs with thin (3.4 µm) and thick (50 µm) Pd-Ag membranes. The effects of the feed temperature (from 450 to 550 °C), pressure (in the range of 2–20 bar), and biogas composition (CH_4_/CO_2_ molar ratios from 1/1 to 7/3) were studied for the thin membrane through the calculation and comparison of several process indicators, namely CH_4_ and CO_2_ conversions, H_2_ yield, H_2_/CO ratio and H_2_ recovery. Estimation of the CO-inhibiting effect on the H_2_ molar flux through the membrane was assessed for a thick membrane. Simulations for a thin Pd-Ag MR show that (i) CO_2_ and CH_4_ conversions and H_2_ yield increase with the feed temperature; (ii) H_2_ yield and average rate of coke formation increase for higher pressures; and (iii) increasing CH_4_/CO_2_ feed molar ratio leads to higher H_2_/CO ratios, but lower H_2_ yields. Moreover, simulations for a thick Pd-Ag MR showed that the average H_2_ molar flux decreases due to the CO inhibiting effect (*ca*. 15%) in the temperature range considered. In conclusion, this work showed that for the considered simulation conditions, the use of an MR leads to the inhibition of the RWGS reaction and improves H_2_ yield, but coke formation and CO inhibition on H_2_ permeation may pose limitations on its practical feasibility, for which proper strategies must be explored.

## 1. Introduction

Biogas is a valuable renewable energy source that can help mitigate greenhouse gas emissions and contribute to climate neutrality [1]. Biogas has been mainly used for combined production of heat and power. It satisfies energy needs in areas not covered by the national grid and provides a clean cooking fuel, preventing the use of solid biomass [2]. In 2020, the global biogas market was valued at around USD 24.03 billion, with the European market representing the major share [3]. Moreover, it is expected that the global market will be valued at around USD 37.02 billion by 2028 [3].

Biomethane results from the upgrading process of biogas, and its production volumes are increasing rapidly [4]. It is expected that its production volumes increase from 3 to 35 bcm by 2030 in the EU as a part of the RePowerEU plan [5,6]. Biomethane is an alternative to natural gas for heat and power generation and as a feedstock to produce high-value chemicals. Furthermore, it allows the reduction in emissions in sectors that are hard to decarbonize like heavy industry and freight transport [2]. It is envisioned that biomethane and hydrogen will contribute to achieving the sub-target of a 14% renewable energy consumption in road and rail transport by 2030, as stipulated in RED II [7].

In the Power-to-Gas (PtG) concept, CO_2_ contained in biogas and previously separated during upgrading can be further valorized into more biomethane, using green hydrogen obtained from water electrolysis [8]. Thus, PtG allows the conversion of electrical energy into chemical energy while boosting biomethane production and avoiding CO_2_ emissions [9].

With the subsidies for electricity production from biogas running out in many countries, including Portugal [10,11], along with the need to adopt sustainable solutions and mitigate the current energy crisis [6], other routes for biogas valorisation are required. Among them, biogas upgrading is a mature option allowing to produce biomethane that can replace natural gas, especially in hard-to-decarbonize sectors. Complementarily, with the growing demand for renewable hydrogen worldwide, dry reforming of methane (DRM) contained in biogas (Equation (1)) [12] is also an interesting valorisation pathway that can unlock biogas potential and create new business models [13]. Alternative renewable hydrogen production routes are increasingly important to fulfil demand, considering that current green hydrogen share is still very low (i.e., 4% of total hydrogen produced worldwide) [14]. Besides injection in the gas grids, several other applications for renewable hydrogen implementation are being considered in the refining, chemical sector and in shipping [4].

The DRM offers the advantage of using two main greenhouse gases, CO_2_ and CH_4_, to produce hydrogen. Furthermore, it provides a route for direct biogas utilization since its main constituents are CO_2_ and CH_4_. Nickel-based catalysts are the most used for DRM because they are the cheapest and offer relatively good activity and selectivity [15]. However, these conventional catalysts usually deactivate due to coke formation [12]. Noble metal catalysts are more resistant to coke formation but are too expensive to be used industrially [15]. In addition, the reverse water gas shift (RWSG) reaction (Equation (2)) can occur in parallel with DRM, which is undesirable because it consumes the hydrogen produced [16]. DRM occurs typically at temperatures between 700 °C and 950 °C because high temperatures lessen the side reactions and coke formation [12]. However, high temperatures can cause catalyst sintering and lead to high operational costs [12].

(1) $$CH_{4}+CO_{2}⇌2CO+2H_{2}\;\;\;\;\Delta H_{r}{}^{298\;K}=247\;\mathrm{kJ}\cdot {\mathrm{mol}}^{-1}$$

(2) $$CO_{2}+H_{2}⇌CO+H_{2}O\;\;\;\;\Delta H_{r}{}^{298\;K}=41.7\;\mathrm{kJ}\cdot {\mathrm{mol}}^{-1}$$

To avoid/minimize these adverse effects, the use of a hydrogen-selective membrane reactor (MR) is envisaged to shift the reaction equilibrium of the DRM reaction (and disfavor the RWGS reaction) through the removal of a product (i.e., H_2_) and to operate at lower temperatures (while obtaining the same conversion as that attained in a fixed-bed reactor at a higher temperature) [16,17]. However, coke formation is still an issue, and new catalysts are still under development [15]. The implementation of MRs contributes to process intensification [18] and provides several advantages such as reduced capital costs (by using smaller devices), improved yield and selectivity and reduced downstream separation costs [19]. 

Thus, the objective of this work is to assess the advantages of using MRs for this application through computational simulation. To this end, a non-isothermal, one-dimensional, steady-state and pseudo-homogeneous plug flow model with axial dispersion is proposed and loaded with suitable reaction kinetics and membrane properties obtained after a literature survey. 

## 2. Computational Methods

A program developed in MATLAB R2015a was employed in this work to simulate the operation of the membrane and traditional reactors. The models were solved using function bvp4c, which solves systems of ordinary differential equations (ODEs) subjected to boundary conditions. The reactors considered were assessed at steady state, and all simulations were carried out using 200 equidistant points along the reactor length. The reactor models employed, and the kinetic and membrane properties considered, are described in the following sections. The local calculations of all physical properties required to solve the reactor models are explained in Supporting Information. The meaning of the variables is presented in the Notation Section.

### 2.1. Traditional and Membrane Reactor Models

The traditional reactor (TR) model considers a fixed-bed tubular reactor packed with a catalyst. The considered feed is a biogas stream without impurities (i.e., binary mixture of CH_4_ and CO_2_). The TR is placed inside a furnace where the temperature is assumed constant and equal to the feed temperature. The model is a non-isothermal, pseudo-homogeneous, steady-state and one-dimensional with axial dispersion [20]. The following assumptions are thus considered in this model:

- Absence of external and internal mass and heat transfer resistances, meaning that $C=C_{s}=C_{b}$ and $T=T_{s}=T_{b}$;
- One-dimensional model across the normalized reactor length (z);
- Porosity of the catalytic bed ($\varepsilon_{b}$) is assumed constant;
- All gases have an ideal behaviour.

Furthermore, the pressure drop along the reactor is described by the Ergun equation.

The ODEs of the mathematical model for the TR are listed in Table 1. Equation (3) represents the partial mass balance for each species i (CO_2_, H_2_, H_2_O, CH_4_, CO, N_2_), and Equation (4) represents the energy balance. The total mass balance is described by Equation (5), and Equation (6) finally describes the momentum balance. The Danckwerts boundary conditions for these ODEs are also listed in Table 1. Equations (7)–(10) describe the boundary conditions in the reactor inlet for z=0, while Equations (11) and (12) describe the boundary conditions in the reactor outlet for z=1.

**Figure 1:**
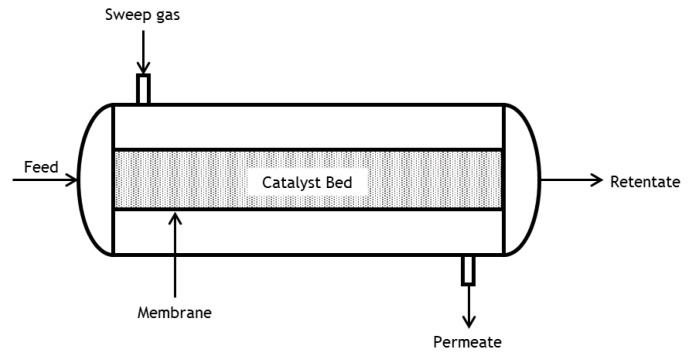
Illustration of the MR configuration.

The membrane reactor (MR) model features a catalytic bed enclosed by a tubular membrane that divides the reactor into two zones, the retentate and the permeate. Feed and sweep gas streams are at the same temperature and have the same flowrate. The catalytic bed is packed in the retentate chamber. The permeate chamber is the annular zone between the membrane and the reactor wall; it is fed with a sweep gas flowing in a co-current mode to the reacting mixture. A pure N_2_ stream is used as sweep gas (though in practice other possibilities can be considered, e.g., steam) to increase the driving force for permeation along the reactor’s length. Figure 1 shows a schematic representation of the MR configuration. The assumptions listed above for the TR model also apply to the MR model. Additionally, plug flow and no pressure drop are assumed in the permeate chamber (due to the absence of a packed bed).

The ODEs that comprise the mathematical model for the retentate zone of the MR are listed in Table 2. Equation (13) represents the partial mass balance for each species i and Equation (14) describes the energy balance for the retentate zone. Total mass and momentum balances for this zone are described by Equations (15) and (16), respectively. The boundary conditions for these ODEs are also listed in Table 2. Equations (17)–(20) describe the boundary conditions in the reactor’s inlet (z=0), while Equations (21) and (22) the boundary conditions in the reactor’s outlet (z=1).

The ODEs that comprise the mathematical model for the permeate zone of the MR are also listed in Table 2. Equation (23) represents the partial mass balance for the species i in the permeate zone, namely hydrogen because only such species was considered to permeate through the selective membrane employed (at a flux $J_{i}$). Equation (24) describes the energy balance for the permeate zone of the MR. Total mass balance is described by Equation (25). The boundary conditions for these ODEs are also listed in Table 2. Equations (26)–(29) describe the boundary conditions in the reactor inlet.

**Table 1:** Mathematical model for the TR.

Ordinary Differential Equations	
Partial Mass Balance for species i:	
$\frac{\varepsilon_{b}\;}{L^{2}}\;\frac{d}{dz}\left(D_{ea}C_{b}\frac{dy_{i,b}}{dz}\right)-\frac{d\left(u_{0}\;C_{i,b}\right)}{L\;dz}+\sum_{j}\rho_{b}\;\alpha_{i,j}\;{{ℜ}^{\prime }}_{j}=0$	(3)
Energy Balance:	
$\frac{1}{L^{2}}\;\frac{d}{dz}\left(\lambda_{ea}\frac{dT_{b}}{dz}\right)-\frac{u_{0}\;\rho_{f}}{L}\;\frac{d\left(T_{b}\;C_{p,f}\right)}{dz}+\sum_{j}\rho_{b}\;\left(-\Delta H\right)\;{{ℜ}^{\prime }}_{j}-\frac{2\;U}{r}\;\left(T_{b}-T_{\infty }\right)=0$	(4)
Total Mass Balance:	
$\frac{d\left(u_{0}\;C_{b}\right)}{L\;\;dz}-\sum_{j}\rho_{b}\sum_{i}(\alpha_{i,j})\;ℜ\prime_{j}=0$	(5)
Momentum Balance:	
$\frac{dP}{dz}=-L\left(150\;\frac{{\left(1-\varepsilon_{b}\right)}^{2}\;u_{f}}{\varepsilon_{b}{}^{3}\;d_{p}{}^{2}}\cdot u_{0}+1.75\;\frac{\left(1-\varepsilon_{b}\right)\;\rho_{f}}{\varepsilon_{b}{}^{3}\;d_{p}}\cdot u_{0}{}^{2}\right)$	(6)
Boundary Conditions	
For z=0	
$\frac{dC_{i,b}}{dz}=-\frac{u_{0}\;L}{\varepsilon_{b}\;D_{ea}}\;\left(C_{i,b}^{\mathrm{in}}-C_{i,b}\right)$	(7)
$\frac{dT_{b}}{dz}=-\frac{u_{0}\;\rho_{f}\;C_{p,f}\;L}{\lambda_{ea}}\;\left(T^{\mathrm{in}}-T_{b}\right)$	(8)
$P=P^{\mathrm{in}}$	(9)
$u_{0}=u_{0}{}^{\mathrm{in}}$	(10)
For z=1	
$\frac{dC_{i,b}}{dz}=0$	(11)
$\frac{dT_{b}}{dz}=0$	(12)

**Table 2:** Mathematical model for the MR.

Retentate Side	
Ordinary Differential Equations	
Partial Mass Balance for species i:	
$\frac{\varepsilon_{b}\;}{L^{2}}\frac{d}{dz}\left(D_{ea}^{R}C_{b}^{R}\frac{dy_{i,b}^{R}}{dz}\right)-\frac{d\left(u_{0}^{R}\;C_{i,b}^{R}\right)}{L\;dz}+\sum_{j}\rho_{b}^{R}\;\alpha_{i,j}\;ℜ\prime_{j}-\frac{2\;\pi \;r^{R}}{A^{R}}\cdot J_{i}=0$	(13)
Energy Balance:	
$\frac{1}{L^{2}}\;\frac{d}{dz}\left(\lambda_{ea}^{R}\frac{dT_{b}^{R}}{dz}\right)-\frac{u_{0}^{R}\;\rho_{f}^{R}}{L}\;\frac{d\left(T_{b}^{R}\;C_{p,f}^{R}\right)}{dz}+\sum_{j}\rho_{b}\;\left(-\Delta H_{j}\right)\;{{ℜ}^{\prime }}_{j}-\frac{2\;\pi \;r^{R}}{A^{R}}\left[U^{R}\;\left(T_{b}^{R}-T_{b}^{P}\right)+\sum_{i}\left(J_{i}\;C_{p,i}^{R}\right)\;\left(T_{b}^{R}-T_{b}^{P}\right)\right]=0$	(14)
Total Mass Balance:	
$\frac{d\left(u_{0}^{R}\;C_{b}^{R}\right)}{L\;\;dz}-\underset{j}{\sum }\rho_{b}^{R}\underset{i}{\sum }(\alpha_{i,j})\;ℜ\prime_{j}+\frac{2\;\pi \;r^{R}}{A^{R}}\;J_{i}=0$.	(15)
Momentum Balance:	
$\frac{dP^{R}}{dz}=-L\left[150\;\frac{{\left(1-\varepsilon_{b}\right)}^{2}\;u_{f}^{R}}{\varepsilon_{b}{}^{3}\;d_{p}{}^{2}}\;u_{0}^{R}+1.75\;\frac{\left(1-\varepsilon_{b}\right)\;\rho_{f}^{R}}{\varepsilon_{b}{}^{3}\;d_{p}}\;{\left(u_{0}^{R}\right)}^{2}\right]$	(16)
Boundary Conditions	
For z=0	
$\frac{dC_{i,b}{}^{R}}{dz}=-\frac{u_{0}{}^{R}\;L}{\varepsilon_{b}\;D_{ea}}\;\left(C_{i,b}^{R,\mathrm{in}}-C_{i,b}^{R}\right)$	(17)
$\frac{dT_{b}{}^{R}}{dz}=-\frac{u_{0}{}^{R}\;\rho_{f}{}^{R}\;C_{p,f}{}^{R}\;L}{\lambda_{ea}}\;\left(T^{R,\mathrm{in}}-T_{b}{}^{R}\right)$	(18)
$P^{R}=P^{R,\;\mathrm{in}}$	(19)
$u_{0}{}^{R}=u_{0}{}^{R,\;\mathrm{in}}$	(20)
For z=1	
$\frac{dC_{i,b}{}^{R}}{dz}=0$	(21)
$\frac{dT_{b}{}^{R}}{dz}=0$	(22)
**Permeate Side**	
Ordinary Differential Equations	
Partial Mass Balance for species i:	
$\frac{d\left(u_{0}^{P}\;C_{i,b}^{P}\right)}{L\;dz}-\frac{2\;\pi \;r^{R}}{A^{P}}\;J_{i}=0$	(23)
Energy Balance:	
$\frac{u_{0}^{P}\;\rho_{f}^{P}}{L}\;\;\frac{d\left(T_{b}^{P}\;C_{p,f}^{P}\right)}{dz}\;\frac{A^{P}}{2\;\pi }=\left[r^{R}\;U^{R}\;\left(T_{b}^{R}-T_{b}^{P}\right)+r^{R}\sum_{i}\left(J_{i}\;C_{p,i}^{P}\right)\;\left(T_{b}^{R}-T_{b}^{P}\right)-r^{P}\;U^{P}\;\left(T_{b}^{P}-T^{\infty }\right)\right]$	(24)
Total Mass Balance:	
$\frac{d\left(u_{0}^{P}\;C_{b}^{P}\right)}{L\;dz}-\frac{2\;\pi \;r^{R}}{A^{P}}\underset{i}{\sum }J_{i}=0$	(25)
Boundary Conditions	
For z=0	
$C_{i,b}^{P}=\frac{y_{i}^{P}\;P^{P}}{R\;T^{P}}$	(26)
$T_{b}^{P}=T^{P,\mathrm{in}}$	(27)
$P^{P}=P^{P,\mathrm{in}}$	(28)
$u_{0}^{P}=u_{0}{}^{P,\mathrm{in}}$	(29)

Finally, the Sieverts’ law, which describes the molar flux of H_2_ through the membrane when the bulk diffusion of atomic hydrogen is the limiting step of permeation, is expressed in Equation (30) [21].

(30) $$J_{H_{2}}=Perm_{H_{2}}^{R}\;[{\left(p_{H_{2}}^{R}\right)}^{0.5}-{\left(p_{H_{2}}^{P}\right)}^{0.5}]$$

### 2.2. Kinetic Equations

The kinetic model implemented in the simulations was the same as that reported by [22]. Those authors determined the kinetics for five relevant reactions over a Ni/Al_2_O_3_ catalyst in the temperature range of 450–650 °C and for a total pressure of 1 bar. The main reactions studied were DRM, RWGS and methane decomposition (MD), described by Equations (1), (2) and (31), respectively. The other two reactions considered were carbon gasification promoted by H_2_O and CO_2_. However, gasification reactions were not considered in our simulations since their reaction rates are negligible in the temperature range considered. This is in agreement with the work done by [23], who reported that in the temperature range of 475–550 °C, the most relevant reactions were DRM, RWGS and MD.

(31) $$CH_{4}⇌C\left(s\right)+2H_{2}\;\;\;\;\Delta H_{r}{}^{298\;K}=74.87\;\mathrm{kJ}\cdot {\mathrm{mol}}^{-1}$$

The reaction rates for DRM, RWGS and MD are expressed by Equations (32), (33) and (34), respectively.

(32) $${ℜ}_{1}=\frac{k_{1}\;K_{{\mathrm{CO}}_{2},1}\;K_{{\mathrm{CH}}_{4},1}\;p_{{\mathrm{CO}}_{2}}\;p_{{\mathrm{CH}}_{4}}}{{\left(1+K_{{\mathrm{CO}}_{2},1}\;p_{{\mathrm{CO}}_{2}}+K_{{\mathrm{CH}}_{4},1}\;p_{{\mathrm{CH}}_{4}}\right)}^{2}}\;\left[1-\frac{{\left(p_{CO}\;p_{H_{2}}\right)}^{2}}{K_{p1}\;p_{{\mathrm{CO}}_{2}}\;p_{{\mathrm{CH}}_{4}}}\right]$$

(33) $${ℜ}_{2}=\frac{k_{2}\;K_{{\mathrm{CO}}_{2},2}\;K_{H_{2},2}\;p_{{\mathrm{CO}}_{2}}\;p_{H_{2}}}{{\left(1+K_{{\mathrm{CO}}_{2},2}\;p_{{\mathrm{CO}}_{2}}+K_{H_{2},2}\;p_{H_{2}}\right)}^{2}}\;\left[1-\frac{\left(p_{CO}\;p_{H_{2}O}\right)}{K_{p2}\;p_{{\mathrm{CO}}_{2}}\;p_{H_{2}}}\right]$$

(34) $${ℜ}_{3}=\frac{k_{3}\;K_{{\mathrm{CH}}_{4},3}\;\left(p_{{\mathrm{CH}}_{4}}-\frac{p_{H_{2}}{}^{2}}{K_{p3}}\right)}{{\left(1+K_{{\mathrm{CH}}_{4},3}\;p_{{\mathrm{CH}}_{4}}+\frac{p_{H_{2}}{}^{1.5}}{K_{H_{2},3}}\right)}^{2}}$$

Additionally, kinetic, equilibrium and adsorption constants employed are described by Equations (35)–(46). Reactions rates (32) to (34) are expressed in mol·kg_cat_^−1^·s^−1^ and partial pressures in bar.

(35) $$k_{1}=1.29\times 10^{6}\;e^{-\frac{102\;065}{R\cdot T}}$$

(36) $$k_{2}=3.5\times 10^{5}\;e^{-\frac{81\;030}{R\cdot T}}$$

(37) $$k_{3}=6.95\times 10^{3}\;e^{-\frac{58\;893}{R\cdot T}}$$

(38) $$K_{CO_{2},1}=2.61\times 10^{-2}\;e^{\frac{37\;641}{R\cdot T}}$$

(39) $$K_{CH_{4},1}=2.60\times 10^{-2}\;e^{\frac{40\;684}{R\cdot T}}$$

(40) $$K_{CO_{2},2}=5.77\times 10^{-1}\;e^{\frac{9\;262}{R\cdot T}}$$

(41) $$K_{H_{2},2}=1.49\;e^{\frac{6\;025}{R\cdot T}}$$

(42) $$K_{CH_{4},3}=2.1\times 10^{-1}\;e^{-\frac{567}{R\cdot T}}$$

(43) $$K_{H_{2},3}=5.18\times 10^{7}\;e^{-\frac{133\;210}{R\cdot T}}$$

(44) $$K_{p1}=6.78\times 10^{14}\;e^{-\frac{259\;660}{R\cdot T}}$$

(45) $$K_{p2}=5.65\times 10^{1}\;e^{-\frac{36\;580}{R\cdot T}}$$

(46) $$K_{p3}=2.98\times 10^{5}\;e^{-\frac{84\;400}{R\cdot T}}$$

### 2.3. Membrane Properties

The MR was simulated considering thin and thick Pd-Ag membranes. Thin supported membranes generally benefit from lowered capital costs because less palladium is used, which allows obtaining higher H_2_ fluxes. However, the synthesis procedure and required equipment can be complex, and their durability, selectivity and sealings challenging. On the other hand, thick self-supported membranes typically are more robust and show infinite selectivity to H_2_, although they might represent a higher initial investment cost. In this regard, it was decided to consider both thin and thick Pd-Ag membranes in the simulation work.

The properties of the thin 3.4 μm Pd-Ag membrane considered were reported by [21]. The membrane’s permeance was evaluated between 400 and 550 °C, since higher temperatures cause membrane instability due to the formation of defects, and for a total pressure difference of 2 bar while keeping the permeate at atmospheric pressure. It was reported that the membrane remained stable for 335 h at 550 °C and its membrane permeance (towards H_2_) dependence on temperature is described by Equation (47) [21].

(47) $$Perm_{H_{2}}^{R}=9.88\times 10^{-3}\;e^{-\frac{8300}{R\cdot T}}$$

The 50 μm thick Pd-Ag dense and self-supported membrane properties reported by [24] were also used in this work. The membrane permeance towards H_2_ was evaluated between 200 and 300 °C and is described by Equation (48). The temperature range considered by [24] is below the range of interest for this application. However, it was verified that the permeance equation of this membrane reasonably describes the H_2_ permeation flux for higher temperatures using the experimental data reported by [25] for a similar but thicker membrane, particularly at 450 °C.

(48) $$Perm_{H_{2}}^{R}=7.36\times 10^{-3}\;e^{-\frac{17\;410}{R\cdot T}}$$

The dense self-supported Pd-Ag membrane permeance equation accounting for the inhibition effect on the membrane permeance due to CO is presented in Equation (49). The beta parameter (proportionality coefficient) and the CO adsorption equilibrium constant necessary to calculate the H_2_ permeance according to the so-called Sieverts–Langmuir equation are presented in Equations (50) and (51), respectively (more details can be found elsewhere, e.g., [24] and [26]).

(49) $$Perm_{H_{2}}^{R}=\left(1-\beta \;\frac{K_{\mathrm{CO}}\;p_{\mathrm{CO}}}{1+K_{\mathrm{CO}}\;p_{\mathrm{CO}}}\right)\;7.36\times 10^{-3}\;e^{-\frac{17\;410}{R\cdot T}}$$

(50) $$\beta =e^{\frac{1\;209}{T}-2.58}$$

(51) $$K_{CO}=e^{\frac{3\;034}{T}-1.12}$$

### 2.4. Simulation Conditions

Table 3 lists the inputs considered in different simulation studies performed in this work.

### 2.5. Performance Indicators

Reactor performance indicators were defined to compare the reactor’s performances when operating at different conditions. The CO_2_ and CH_4_ conversions were defined according to Equation (52). In addition, the yield of H_2_ was also considered since H_2_ is the desired product. The yield of H_2_ is defined according to Equation (53), as reported by [23]. 

The H_2_/CO ratio considering the total H_2_ produced was defined by Equation (54) since it helps to assess which reactions are predominant and the quality of the syngas obtained. Finally, H_2_ recovery allows to evaluate the efficiency of the separation (Equation (55)).

(52) $$X_{i}=\frac{F_{i}^{\mathrm{in}}-F_{i}^{\mathrm{out}}}{F_{i}^{\mathrm{out}}}\times 100\;\;\;\;i={\mathrm{CH}}_{4},{\;\mathrm{CO}}_{2}$$

(53) $$Y_{H_{2}}=\frac{F_{H_{2}}^{\mathrm{out}}}{2\cdot F_{{\mathrm{CH}}_{4}}^{\mathrm{in}}}\times 100$$

(54) $$\frac{H_{2}}{CO}=\frac{F_{H_{2}}^{\mathrm{out}}}{F_{\mathrm{CO}}^{\mathrm{out}}}\times 100$$

(55) $$Rec_{H_{2}}=\frac{F_{H_{2}}^{P}}{F_{H_{2}}^{P}+F_{H_{2}}^{R}}\times 100$$

## 3. Results and Discussion

### 3.1. Kinetic Model Validation

The results obtained experimentally by [22] were compared with simulated results to validate the kinetic model. The fixed-bed dimensions, catalyst parameters and experimental conditions used by those authors to determine the kinetic model are shown in Table 4.

**Figure 2:**
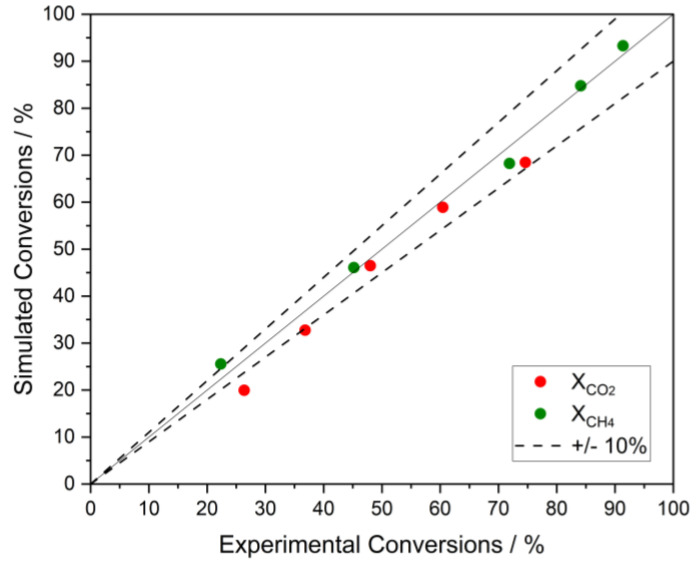
Parity plot comparing the experimental CH_4_ and CO_2_ conversions obtained by [22] with simulated values.

The reactor used by such authors was simulated using the same operating conditions and the TR model described in Section 2.1. Figure 2 shows the parity plot comparing CH_4_ and CO_2_ conversions obtained in simulations with those obtained experimentally by the authors. The figure shows that the maximum difference between the experimental and simulated conversions is approximately 10%, which means that this kinetic model was satisfactorily validated.

### 3.2. Membrane Reactor Simulations

Simulations of an MR were performed using the properties of Pd-Ag membranes presented in Section 2.3. Different operating conditions were used to study the influence of temperature, pressure, biogas composition and CO inhibition on the MR performance (*cf*. Table 3).

#### 3.2.1. Effect of Temperature

**Figure 3:**
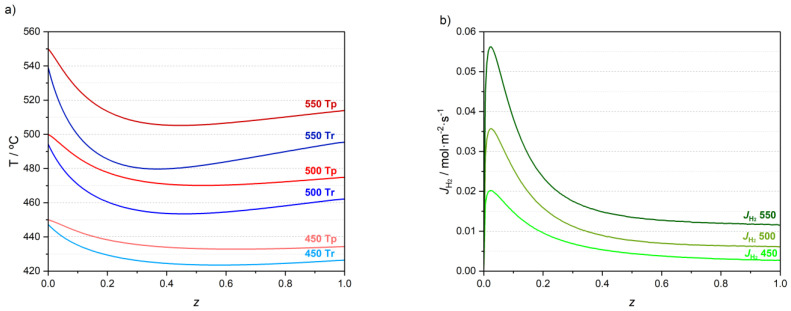
Temperature profiles for the retentate (T_r_) and permeate (T_p_) zones of the reactor (**a**) and permeation fluxes of hydrogen (**b**) when operating the MR with feed temperatures of 450, 500 and 550 °C.

The temperature and H_2_ permeation flux profiles for the three simulations performed using feed temperatures of 450, 500 and 550 °C are represented in Figure 3. Additional mole fraction plots are available in Supporting Information (Figures S1 and S2).

Figure 3a shows temperature profiles on permeate and retentate zones for the three simulations. As previously mentioned, reactions occur in the retentate chamber. The temperature in the retentate chamber initially drops because all the reactions are endothermic. However, the heat transferred from the permeate zone surpasses the heat consumed by the reactions around *z* = 0.6 for the simulation employing a feed temperature of 450 °C. Consequently, the retentate zone temperature increases for *z* > 0.6. The temperature in the permeate zone also decreases initially due to the heat transferred to the retentate zone and to the cooler H_2_ that permeates through the membrane. However, the temperature in the permeate zone increases for z > 0.6 as well, for the simulation at 450 °C. Therefore, the heat transferred by the reactor wall to the permeate zone is higher than the heat transferred to the retentate zone and to the permeated H_2_. For higher feed temperatures, the profiles are more pronounced, and the reactor temperature starts decreasing steadily closer to the reactor inlet before increasing again around *z* = 0.5 and *z* = 0.4 for a 500 °C and 550 °C feed temperature, respectively.

Figure 3b shows that the H_2_ molar flux through the membrane increases sharply and reaches a maximum near the reactor inlet for all simulations. Afterwards, the H_2_ flux decreases mainly because of the lower driving force. Still, the flux is always positive, which means that H_2_ is always permeating from the retentate to the permeate chamber along the entire reactor length. Figure 3b also shows that the H_2_ permeation molar flux increases with the feed temperature, because it is an activated process and because more hydrogen is formed. The permeation molar flux of hydrogen for a feed temperature of 550 °C is threefold higher than that for a feed temperature of 450 °C.

**Figure 4:**
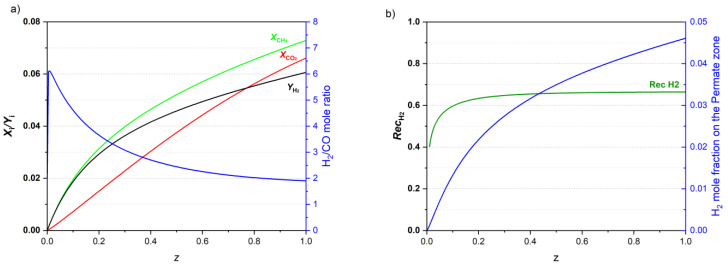
(**a**) CH_4_ and CO_2_ conversions, H_2_ yield and H_2_/CO mole ratio profiles, and (**b**) H_2_ recovery and H_2_ mole fraction profile on the permeate zone along the reactor, operating with a feed temperature of 450 °C.

CH_4_ and CO_2_ conversions, H_2_ yield, and total H_2_/CO ratio profiles for a feed temperature of 450 °C are presented in Figure 4a, while H_2_ recovery and mole fraction of H_2_ in the permeate zone are shown in Figure 4b.

Figure 4a shows that conversions and H_2_ yield continuously increase along the reactor length. However, the increase in these performance indicators is more noticeable closer to the reactor inlet due to the thermodynamic equilibrium of all the reactions (with higher concentrations of reactants and smaller concentrations of products), and because of the higher temperatures in the first fraction of the reactor, closer to its inlet (Figure 3). The H_2_/CO ratio increases considerably close to the reactor inlet since initially the fraction of these components is zero. Then, the ratio declines along the reactor length, reaching a value of 1.9 at the reactor end.

H_2_ is the only species that permeates through the membrane and is diluted due to the use of N_2_ as sweep gas, as seen in Figure 4b. The hydrogen mole fraction in the permeate is quite small (i.e., 4.6%), although the H_2_ recovery achieved is 66%.

Table 5 lists the performance indicators obtained in the simulations performed to study the effect of the feed temperature.

Table 5 shows that CH_4_ conversion and H_2_ yield increase significantly with the temperature due to the improved kinetics and the removal of H_2_ by permeation (i.e., a product of DRM and MD reactions). The H_2_/CO ratio is very high for all simulations, because the average rate of Reaction 3 (MD) is higher than the average rate of Reaction 2 (RWGS) (*cf*. Table 6). The H_2_ recovery remains nearly constant for the different temperatures (Table 5); however, due to the higher H_2_ yields (the amount of H_2_ that permeated increased nearly threefold in the range of 450–550 °C), a higher amount of H_2_ is recovered at higher temperatures. 

Table 6 shows that average rate of the DRM reaction (R1) is the lowest among the three reactions, which is undesirable since it is the main reaction.

#### 3.2.2. Effect of Pressure

The pressure effect on MR performance was studied by changing the feed pressure while keeping all the other operating conditions constant. The operating conditions considered are listed in Table 3.

Five simulations were performed to evaluate the effect of the feed pressure on MR performance. The figures obtained in all simulations showed similar patterns as those shown in the previous section and are available in Supporting Information (Figures S3–S6), while the performance indicators are summarized in Table 7.

The H_2_ recovery increases substantially with the total pressure due to the higher driving force for permeation (*cf*. Equation (30)). Table 7 also shows that the conversion of CH_4_ increases with pressure, while the conversion of CO_2_ decreases. Hence, the higher H_2_ yields and H_2_/CO ratios for higher feed pressures (Table 7) are a consequence of the promotion of Reaction 3 (MD), evidencing that coke formation increases with pressure, as commonly observed for Ni-based catalysts. This is supported by the average rates of reactions listed in Table 8, where is shown that the average reaction rate of MD (R3) is superior to that of the DRM (R1) and RWGS (R2).

Usually, DRM only allows the production of syngas with low H_2_/CO ratios (close to 1) which is preferred for oxygenated chemicals and liquid hydrocarbons production through the Fischer–Tropsch synthesis [27,28]. However, with the use of an MR, it is possible to obtain high-grade syngas with a H_2_/CO ratio close to two that can be used to produce methanol and has potential applications in Fischer–Tropsch operations for the production of long hydrocarbons [29,30]. Syngas with higher H_2_/CO ratios can be used in single-step production of dimethyl ether [31]. 

The increase in rate of Reaction 3 (MD) combined with the decrease in rate of Reaction 2 (RWGS) by increasing the total pressure explains the H_2_/CO ratios reported above. It is also possible to observe that the average rate of Reaction 2 (RWGS) is significantly lower than the average rate of Reaction 1 (DRM) for higher pressures because more H_2_ permeates through the membrane, thus limiting the extension of the RWGS reaction.

#### 3.2.3. Effect of Biogas Composition

The effect of biogas composition on the MR performance was studied by changing the CH_4_/CO_2_ inlet molar ratio. In these simulations, the mass of catalyst and, consequently, the gas hourly space velocity (*GHSV*) were changed while keeping the ratio between the catalyst mass and the inlet CH_4_ molar flowrate equal to 1.08 g_cat_·h·mol_CH4_^−1^ in all simulations. The reactor dimensions, catalyst parameters and operating conditions employed are presented in Table 3 (except *W*). A total pressure of 2 bar was chosen in these simulations because the difference between CH_4_ and CO_2_ conversions is the smallest (*cf*. Table 7), which means that the occurrence of Reaction 3 (MD) is minimized.

**Figure 5:**
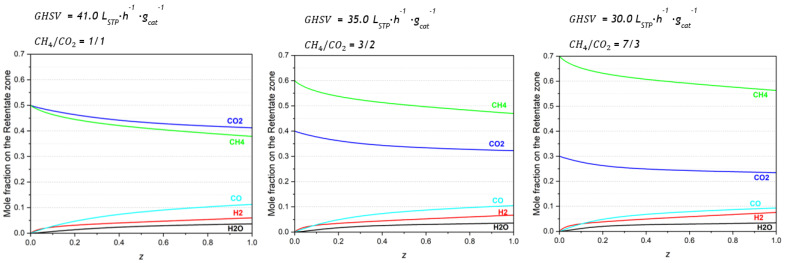
Mole fraction profiles in the retentate zone along the normalized reactor length for different biogas compositions.

Three simulations were performed to evaluate the effect of biogas composition in the MR performance. The mole fraction profiles in the retentate zone are presented in Figure 5. Additional figures are available in Supporting Information (Figures S7 and S8).

The mole fraction profiles along the retentate length show that the outlet mole fraction of CO decreases while the H_2_ fraction increases with the increase in the CH_4_ fraction in the feed stream. For a 1/1 CH_4_/CO_2_ inlet molar ratio, the CO and H_2_ outlet mole fractions are approximately 11% and 6%, respectively, while for a 7/3 CH_4_/CO_2_ inlet, they are approximately 9% and 8%.

**Figure 6:**
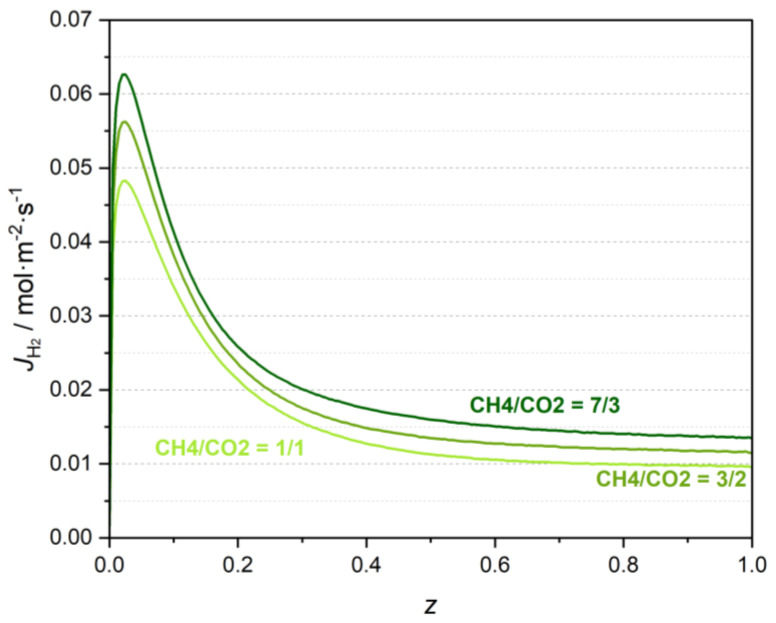
H_2_ molar flux along the normalized reactor length for different biogas compositions.

Figure 6 presents the H_2_ molar flux profiles obtained considering different biogas compositions. It shows that the permeated H_2_ molar flux also increases with the CH_4_/CO_2_ inlet molar ratio.

To evaluate the effect of biogas feed composition, the performance indicators were calculated for the three compositions, and the results are presented in Table 9. It shows that the H_2_/CO ratio increases and the H_2_ yield slightly decreases for biogas feeds richer in CH_4_. The H_2_/CO ratio increased *ca.* 56% from the feed CH_4_/CO_2_ ratio of 1/1 to the 7/3 feed ratio, while the H_2_ yield only decreased *ca.* 11%. The reason for increasing CO_2_ conversions is the higher *W*/*F*_CO2_ ratio for feeds richer in CH_4._ In addition, the recovery remained constant, which means that the CH_4_/CO_2_ inlet ratio does not affect the efficiency of the separation. Still, the permeate H_2_ flowrate increased *ca.* 30% (from the feed CH_4_/CO_2_ ratio of 1/1 to the 7/3 feed ratio) due to the increase in the total amount of H_2_ produced.

#### 3.2.4. Effect of CO and Membrane Thickness

Simulations of the MR were also performed using a thick Pd-Ag dense membrane presented in Section 2.3. The advantage of simulating such an MR is the availability of the Sieverts–Langmuir equation parameters in the literature, which allows the study of the CO inhibiting effect on the H_2_ permeation. Such data are not available for the thin membrane, wherein the CO inhibiting effect was discarded.

**Figure 7:**
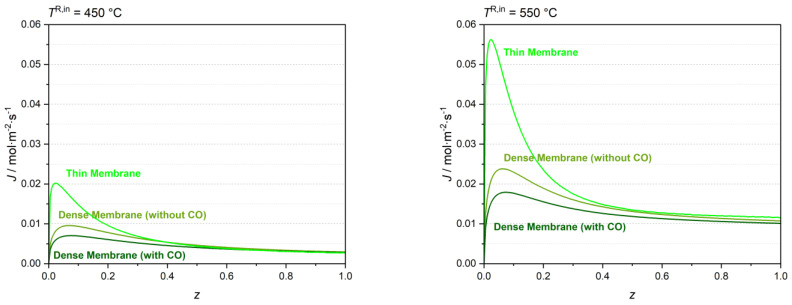
H_2_ molar flux profiles for the MR with thin and dense membranes, with and without considering the CO inhibiting effect and operating with feed temperatures of 450 °C and 550 °C.

The MR was simulated using a feed temperature of 450 °C and 550 °C with and without considering the CO effect on the H_2_ molar flux (i.e., considering the Sieverts–Langmuir or the Sieverts equation, respectively). The H_2_ molar flux profiles obtained with both dense and thin Pd-Ag membranes at 450 °C and 550 °C are presented in Figure 7.

The average molar flux reduction (i.e., calculated using the average H_2_ permeation fluxes along the membrane) for the MR with the dense Pd-Ag membrane considering the CO adsorption on the membrane surface is approximately 17% for a 450 °C feed temperature, and 14% for a 550 °C feed temperature. Thus, the inhibiting effect of CO on the membrane flux is significant at the temperature range considered, particularly at 450 °C where CO adsorption on the metallic membrane is more significant. The figure shows that the H_2_ molar flux is, however, two times higher for a feed temperature of 550 °C when considering the denser membrane. The H_2_ molar flux is considerably higher for the thin membrane. 

To evaluate the effect of the CO inhibiting effect on the MR operation, the performance indicators were calculated and the results for the 550 °C feed temperature are presented in Table 10. The table shows that when accounting for the CO inhibiting effect, the H_2_ recovery decreases slightly (3%, absolute variation). In addition, the H_2_ yield also slightly decreases because the shift in the forward direction of Reactions 1 (DRM) and 3 (MD) due to H_2_ permeation is less pronounced. For the same reason, with the CO effect on the membrane properties, the equilibrium of the forward Reaction 2 (RWGS) should not be inhibited so extensively, increasing CO_2_ conversion.

### 3.3. Comparison between Pd-Ag Thin Membrane Reactor and Traditional Reactor

In this section, TR and MR with a Pd-Ag thin membrane are compared at similar operating conditions. The selected feed temperature was 550 °C because the advantage of adding the membrane is more noticeable at this temperature as shown previously. The feed pressure of 2 bar was chosen because the CH_4_ and CO_2_ conversions are closest at this pressure, minimizing side reactions; finally, the chosen CH_4_/CO_2_ inlet ratio molar ratio was 3/2, which is a compromise between the highest H_2_ yield and the highest H_2_/CO ratio.

To compare the TR and MR operations, performance indicators were calculated for both simulations; they are presented in Table 11. The detailed figures for the MR and TR simulations considered are presented in Supporting Information (Figures S2 and S9).

Table 11 shows that CH_4_ and CO_2_ conversions are closer for the MR, which indicates that the side reactions are minimized. Although the CO_2_ conversion is lower for the MR, the H_2_ yield is significantly higher (increase of 66%). The H_2_/CO ratio is also quite higher for the membrane device (increase of 83%). This indicates that Reaction 3 (MD) is favored in this reactor (*cf*. Table 12), which is, however, undesired due to coke formation. The use of other catalysts could solve this problem, but they are still under development [15]. Another option would be to add steam to the feed stream since it mitigates coke formation [12].

### 3.4. Membrane Reactor Design Considerations—Perspectives for Future Work

In this work, it was assumed that the feed and sweep gas streams delivered to the MR were at the same temperature and flow rate. However, it could also be interesting to study the effect of the sweep gas temperature. Indeed, since the DRM is an endothermic reaction, it may be beneficial to deliver the sweep gas at a temperature above that of the feed stream when considering a catalyst packing in the lumen of the membrane. Alternatively, one can consider placing the catalyst in the annular section and collecting the permeate in the lumen side, although catalyst loading/unloading in such configuration would be more challenging in practice.

The use of sweep gas aims to hold the H_2_ partial pressure in the permeate chamber low (ideally zero) along the MR length, basically to counterbalance the enrichment due to its permeation, which is particularly relevant for thin membranes (i.e., for higher permeation fluxes). However, the use of a sweep gas dilutes H_2_ and so an additional separation step is required. Therefore, the sweep gas type, its flow rate and feed mode (co-current or counter-current) should be carefully selected so that the sweep does not poison nor permeate through the membrane, nor the subsequent H_2_ purification step is difficult. Alternatively, vacuum can be used to increase the driving force for permeation without diluting H_2_, or the use of a sweep gas/vacuum be discarded for techno-economic reasons. 

Finally, in this work, it was considered that H_2_ permeation was controlled by hydrogen bulk diffusion (i.e., following Sieverts’ law (*n* = 0.5)) based on data collected from the literature for both membranes. However, the occurrence of deviations to Sieverts’ law is also frequently reported in the literature due to additional mass transport resistances, for instance, in the porous support or due to the occurrence of concentration polarization (especially in the case of thin membranes). Hence, to conclude the discussion about the true potential of using Pd-Ag MRs for low-temperature dry reforming, all the considerations mentioned above should be further addressed, as well as the membrane and catalyst deactivation by coke and possible regeneration strategies.

## 4. Conclusions

The results for the MR simulated with a thin Pd-Ag membrane showed that CO_2_ and CH_4_ conversions and H_2_ yield notably increased with the feed temperature, while H_2_ recovery was somewhat constant (*ca.* 67%) in the temperature range studied (450–550 °C). With the feed pressure increase, in the range of 2–20 bar, the CH_4_ conversion increases from 20.3% to 25.8%, and the CO_2_ conversion decreases from 17.9% to 9.5%, enlarging the gap between them. Therefore, the reaction rate of MD was higher for higher pressures, increasing coke production. Higher H_2_ recoveries were achieved for higher feed pressures (up to 96% at 20 bar) due to the increasing driving force, while increasing the CH_4_/CO_2_ feed molar ratio led to higher H_2_/CO ratios (up to 2.49 for CH_4_/CO_2_ = 7/3) but slightly lower H_2_ yields (minimum of 16.5 % for CH_4_/CO_2_ = 7/3).

The simulation of an MR with a dense Pd-Ag membrane allowed the study of the CO inhibiting effect on the H_2_ molar flux through the membrane. The results for this reactor show that the average H_2_ molar flux significantly decreases (i.e., *ca.* 15%) in the temperature range considered.

Finally, the results obtained for the comparison of the TR and MR performance show that the RWGS can be inhibited and that the H_2_ yield and the H_2_/CO ratio increase in the MR. However, the average rate of coke formation is also higher for the MR, particularly at high pressure, which can in practice be minimized with the appropriate choice of a catalyst to employ and/or by conjugating dry with steam reforming. 

## Figures and Tables

**Table 3:** Simulation inputs considered for the different studies.

Reactor Dimensions and Catalyst Parameters														
Membrane thickness/µm		$D^{R}/m$		$D^{P}/m$			L/m			W/mg			$d_{p}/\mu m$	
0 (i.e., TR)		0.010		n.a.			0.10			1000			320	
3.4				0.015										
50														
Operating Conditions Used to Study the Temperature Effect														
Membrane thickness (µm)	$T^{R,\mathrm{in}}/{}^{\circ}C$		$P^{R,\mathrm{in}}/\mathrm{bar}$		$\frac{P^{R,\mathrm{in}}}{P^{P,\mathrm{in}}}$	$GHSV/L_{\mathrm{STP}}\cdot h^{-1}\cdot g_{\mathrm{cat}}{}^{-1}$					$CH_{4}/CO_{2}\;Inlet\;molar\;ratio$			
3.4	450–550		2		2	35.0					3/2			
Operating Conditions Used to Study the Pressure Effect														
Membrane thickness (µm)	$T^{R,\mathrm{in}}/{}^{\circ}C$		$P^{R,\mathrm{in}}/\mathrm{bar}$		$\frac{P^{R,\mathrm{in}}}{P^{P,\mathrm{in}}}$	$GHSV/L_{\mathrm{STP}}\cdot h^{-1}\cdot g_{\mathrm{cat}}{}^{-1}$					$CH_{4}/CO_{2}\;Inlet\;molar\;ratio$			
3.4	550		2–20		2–20	35.0					3/2			
Operating Conditions Used to Study the Biogas Composition Effect														
Membrane thickness (µm)	$T^{R,\mathrm{in}}/{}^{\circ}C$		$P^{R,\mathrm{in}}/\mathrm{bar}$		$\frac{P^{R,\mathrm{in}}}{P^{P,\mathrm{in}}}$	$GHSV/L_{\mathrm{STP}}\cdot h^{-1}\cdot g_{\mathrm{cat}}{}^{-1}$					$CH_{4}/CO_{2}\;Inlet\;molar\;ratio$			
3.4	550		2		2	41.0		35.0	30.0		1/1	3/2		7/3
Operating Conditions Used to Study the CO Inhibiting Effect on the H_2_ Flux														
Membrane thickness (µm)	$T^{R,\mathrm{in}}/{}^{\circ}C$		$P^{R,\mathrm{in}}/\mathrm{bar}$		$\frac{P^{R,\mathrm{in}}}{P^{P,\mathrm{in}}}$	$GHSV/L_{\mathrm{STP}}\cdot h^{-1}\cdot g_{\mathrm{cat}}{}^{-1}$					$CH_{4}/CO_{2}\;Inlet\;molar\;ratio$			
50	450	550	2		2	35.0					3/2			
Operating Conditions Used to Compare the Performance of the TR and MR														
Membrane thickness (µm)	$T^{R,\mathrm{in}}/{}^{\circ}C$		$P^{R,\mathrm{in}}/\mathrm{bar}$		$\frac{P^{R,\mathrm{in}}}{P^{P,\mathrm{in}}}$	$GHSV/L_{\mathrm{STP}}\cdot h^{-1}\cdot g_{\mathrm{cat}}{}^{-1}$					$CH_{4}/CO_{2}\;Inlet\;molar\;ratio$			
0 (i.e., TR)	550		2		n.a.	35.0					3/2			
3.4					2									

**Table 4:** Fixed-bed reactor dimensions, catalyst parameters and experimental conditions [22].

D/m	L/m	W/mg	$d_{p}/\mu m$	T/°C	P/bar	GHSV $/L_{\mathrm{STP}}\cdot h^{-1}\cdot g_{\mathrm{cat}}{}^{-1}$	$CH_{4}/CO_{2}/N_{2}\;\mathrm{Inlet}\mathrm{Molar}\mathrm{Ratio}$
0.008	0.031	200	320	450–650	1	16	1/1/8

**Table 5:** Performance indicators calculated for the three feed temperatures considered.

$T^{R,in}/{}^{\circ}C$	$X_{CH_{4}}$	$X_{CO_{2}}$	$Y_{H_{2}}$	$\frac{H_{2}}{CO}$	$Rec_{H_{2}}$
450	7.3%	6.6%	6.1%	1.90	66%
500	12.9%	12.1%	10.7%	1.84	67%
550	20.3%	17.9%	17.3%	1.94	67%

**Table 6:** Average rate of Reaction 1 (DRM) along the reactor and normalized average rates of Reactions 2 (RWGS) and 3 (MD) calculated for the temperatures considered.

$T^{in}/{}^{\circ}C$	$\bar{{ℜ}_{1}}/mol\cdot kg_{\mathrm{cat}}{}^{-1}\cdot s^{-1}$	$\bar{{ℜ}_{2}}/\bar{{ℜ}_{1}}$	$\bar{{ℜ}_{3}}/\bar{{ℜ}_{1}}$
450	5.05 × 10^−3^	1.24	1.82
500	9.27 × 10^−3^	1.22	1.74
550	1.51 × 10^−3^	1.03	1.65

**Table 7:** Performance indicators calculated for the feed pressures considered.

$P^{R,in}/\mathrm{bar}$	$X_{CH_{4}}$	$X_{CO_{2}}$	$Y_{H_{2}}$	$\frac{H_{2}}{CO}$	$Rec_{H_{2}}$
2	20.3%	17.9%	17.3%	1.94	67%
5	22.8%	14.0%	20.6%	2.90	85%
10	24.6%	11.4%	23.1%	3.81	92%
15	25.4%	10.2%	24.2%	4.32	95%
20	25.8%	9.5%	24.8%	4.61	96%

**Table 8:** Average rate of Reaction 1 (DRM) along the reactor and normalized average rates of Reactions 2 (RWGS) and 3 (MD) calculated for the feed pressures considered.

$P^{in}/\mathrm{bar}$	$\bar{{ℜ}_{1}}/\mathrm{mol}\cdot kg_{\mathrm{cat}}{}^{-1}\cdot s^{-1}$	$\bar{{ℜ}_{2}}/\bar{{ℜ}_{1}}$	$\bar{{ℜ}_{3}}/\bar{{ℜ}_{1}}$
2	1.51 × 10^−2^	1.03	1.65
5	1.25 × 10^−2^	0.91	2.56
10	1.16 × 10^−2^	0.68	3.17
15	1.14 × 10^−2^	0.52	3.40
20	1.14 × 10^−2^	0.42	3.48

**Table 9:** Performance indicators calculated for the CH_4_/CO_2_ inlet molar ratios considered.

$CH_{4}/CO_{2}\;Inlet\;Molar\;Ratio$	$X_{CH_{4}}$	$X_{CO_{2}}$	$Y_{H_{2}}$	$\frac{H_{2}}{CO}$	$Rec_{H_{2}}$
1/1	22.2%	15.3%	18.5%	1.60	67%
3/2	20.3%	17.9%	17.3%	1.94	67%
7/3	19.1%	21.3%	16.5%	2.49	67%

**Table 10:** Performance indicators calculated with and without considering the CO inhibiting effect on the membrane flux for a 550 °C feed temperature.

Reactor	$X_{CH_{4}}$	$X_{CO_{2}}$	$Y_{H_{2}}$	$\frac{H_{2}}{CO}$	$Rec_{H_{2}}$
without CO effect	18.6%	18.6%	15.1%	1.69	58%
with CO effect	18.1%	18.8%	14.5%	1.62	55%

**Table 11:** Performance indicators calculated for TR and MR simulations.

Reactor	$X_{CH_{4}}$	$X_{CO_{2}}$	$Y_{H_{2}}$	$\frac{H_{2}}{CO}$	$Rec_{H_{2}}$
Traditional	15.7%	22.7%	10.4%	1.06	-
Membrane	20.3%	17.9%	17.3%	1.94	67%

**Table 12:** Average rate of Reaction 1 (DRM) along the reactor and normalized average rates of Reactions 2 (RWGS) and 3 (MD) calculated for the TR and MR simulations.

Reactor	$\bar{{ℜ}_{1}}/\mathrm{mol}\cdot kg_{\mathrm{cat}}{}^{-1}\cdot s^{-1}$	$\bar{{ℜ}_{2}}/\bar{{ℜ}_{1}}$	$\bar{{ℜ}_{3}}/\bar{{ℜ}_{1}}$
Traditional	1.14 × 10^−2^	2.40	1.61
Membrane	1.51 × 10^−2^	1.03	1.65

## Data Availability

The data presented in this study are available on request from the corresponding author.

## Supplementary Materials

The following supporting information can be downloaded at: https://www.mdpi.com/article/10.3390/membranes13070630/s1, Figure S1: Mole fractions on the retentate (a) and permeate (b) zones, temperature (c), and permeated molar flux of hydrogen (d) along the reactor, operating with a feed temperature of 500 °C; Figure S2: Mole fractions on the retentate (a) and permeate (b) zones, temperature (c), and permeated molar flux of hydrogen (d) along the reactor, operating with a feed temperature of 550 °C; Figure S3: Mole fractions on the retentate (a) and permeate (b) zones, temperature (c), and permeated molar flux of hydrogen (d) along the reactor, operating with a feed pressure of 5 bar; Figure S4: Mole fractions on the retentate (a) and permeate (b) zones, temperature (c), and permeated molar flux of hydrogen (d) along the reactor, operating with a feed pressure of 10 bar; Figure S5: Mole fractions on the retentate (a) and permeate (b) zones, temperature (c), and permeated molar flux of hydrogen (d) along the reactor, operating with a feed pressure of 15 bar; Figure S6: Mole fractions on the retentate (a) and permeate (b) zones, temperature (c), and permeated molar flux of hydrogen (d) along the reactor, operating with a feed pressure of 20 bar; Figure S7: Mole fractions on the retentate (a) and permeate (b) zones, temperature (c), and permeated molar flux of hydrogen (d) along the reactor, operating with a 1/1 CH_4_/CO_2_ inlet molar ratio and a GHSV of 41.0 L_STP_·h^−1^·gcat^−1^; Figure S8: Mole fractions on the retentate (a) and permeate (b) zones, temperature (c), and permeated molar flux of hydrogen (d) along the reactor, operating with a 7/3 CH_4_/CO_2_ inlet molar ratio and a GHSV of 30.0 L_STP_·h^−1^·gcat^−1^; Figure S9: Mole fractions (a), temperature (b), and molar flowrate (c) along the traditional reactor length, Mole fractions (**a**), temperature (b), and molar flowrate (c) along the traditional reactor length, operating with a feed temperature of 550 °C, a feed pressure of 2 bar and a 3/2 CH_4_/CO_2_ inlet molar ratio; Table S1: Thermodynamic properties for the species considered; Table S2: Sum of the atomic diffusion volumes for the compounds considered; Table S3: Coefficients used to estimate $C_{p,i}$; Table S4: Coefficients to calculate the viscosity of the compounds considered.

## Nomenclature

Notation		
A	Cross section area	m^2^
C	Molar concentration	mol·m^−3^
$C_{p}$	Specific heat capacity	J·K^−1^·kg^−1^
$d_{p}$	Catalyst particle diameter	m
D	Diameter	m
$D_{ea}$	Mass axial dispersion coefficient	m^2^·s^−1^
GHSV	Total gas hourly space velocity	L_STP_·h^−1^·g_cat_^−1^
H	Enthalpy	J·mol^−1^
J	Molar flux	mol·m^−2^·s^−1^
$k_{i}$	Kinetic constant of reaction *i*	mol·kg_cat_^−1^·s^−1^
$K_{CO}$	CO adsorption constant	bar^−1^
$K_{CH_{4}}$	CH_4_ adsorption constant	bar^−1^
$K_{CO_{2}}$	CO_2_ adsorption constant	bar^−1^
$K_{H_{2},2}$	H_2_ adsorption constant for the RWGS reaction	bar^−1^
$K_{H_{2},3}$	H_2_ adsorption constant for the MD reaction	bar^1.5^
$K_{p1}$	Equilibrium constant for the DRM reaction	bar^2^
$K_{p2}$	Equilibrium constant for the RWGS reaction	dimensionless
$K_{p3}$	Equilibrium constant for the MD reaction	bar
L	Reactor length	m
p	Partial pressure	Pa
P	Total pressure	Pa
Perm	Permeance	mol·m^−2^·s^−1^·Pa^−0.5^
r	Reactor radius	m
ℜ	Reaction rate	mol·kg_cat_^−1^·s^−1^
$Rec_{H_{2}}$	H_2_ recovery	dimensionless
T	Temperature	K
$u_{0}$	Superficial gas velocity	m·s^−1^
U	Global Coefficient of heat transfer	W·m^−2^·K^−1^
W	Catalyst mass	kg
X	Conversion	dimensionless
y	Molar fraction	dimensionless
$Y_{H_{2}}$	H_2_ yield	dimensionless
z	Dimensionless axial coordinate	dimensionless
Greek Letters		
α	Stoichiometric coefficient	dimensionless
β	Proportionality constant	dimensionless
ε	Porosity	dimensionless
$\lambda_{ea}$	Heat axial dispersion coefficient	W·m^−1^·K^−1^
μ	Viscosity	Pa·s
ρ	Density	kg·m^−3^
Indexes		
0	At the first point inside the reactor	
∞	At the furnace	
1	Regarding the DRM reaction	
2	Regarding the RWGS reaction	
3	Regarding the MD reaction	
in	Reactor inlet	
out	Reactor outlet	
b	Bulk	
f	Fluid	
i	Regarding compound *i*	
j	Regarding reaction *j*	
P	Regarding the permeate zone	
R	Regarding the retentate zone	
r	Reaction	
s	Catalyst surface	
Acronyms		
DRM	Dry Reforming of Methane	
EBA	European Biogas Association	
EU	European Union	
IEA	International Energy Agency	
IRENA	International Renewable Energy Agency	
MD	Methane Decomposition	
MR	Membrane Reactor	
ODE	Ordinary Differential Equation	
PtG	Power to Gas	
RED II	Renewable Energy Directive II	
RWGS	Reverse Water Gas Shift	
TR	Traditional Reactor	
