# Supplementary material for: Use of Pd-Ag Membrane Reactors for Low-Temperature Dry Reforming of Biogas—A Simulation Study
Source: Membranes (Basel). 2023 Jun 29;13(7):630. doi: 10.3390/membranes13070630 (PMC10383273; doi:10.3390/membranes13070630)
Supplement: Supplementary file 1 [file membranes-13-00630-s001.zip › membranes-2374395-supplementary.pdf]

*Supplementary Materials*

# Use of Pd-Ag Membrane Reactors for Low-Temperature Dry Reforming of Biogas—A Simulation Study

**Matilde Albano [1,2], Luís M. Madeira [3,4] and Carlos V. Miguel [2,*]**

[1] Department of Chemical Engineering, Faculty of Engineering, University of Porto, Rua Dr. Roberto Frias, 4200-465 Porto, Portugal
[2] Fraunhofer Portugal AWAM—Research Center for Smart Agriculture and Water Management, Régia Douro Park—Parque de Ciência e Tecnologia, 5000-033 Vila Real, Portugal
[3] LEPABE—Laboratory for Process Engineering, Environment, Biotechnology and Energy, Faculty of Engineering, University of Porto, Rua Dr. Roberto Frias, 4200-465 Porto, Portugal
[4] ALiCE—Associate Laboratory in Chemical Engineering, Faculty of Engineering, University of Porto, Rua Dr. Roberto Frias, 4200-465 Porto, Portugal
* Correspondence: carlos.miguel@awam.fraunhofer.pt; Tel.: +351-220430300; Fax: +351-226005029

**Nomenclature**

| | | |
|---|---|---|
| $A_{i,j}$ | Binary interaction | |
| $C_p$ | Specific heat capacity | $J\cdot K^{-1}\cdot kg^{-1}$ |
| $D_{ea}$ | Mass axial dispersion coefficient | $m^2\cdot s^{-1}$ |
| $D_m$ | Molecular diffusion | $m^2\cdot s^{-1}$ |
| $M_i$ | Molecular Weight | $g\cdot mol^{-1}$ |
| $P_{c,i}$ | Critical Pressure | bar |
| $T_{c,i}$ | Critical Temperature | K |
| $d_p$ | Catalyst particle diameter | m |
| $k^{shell}$ | Shell thermal conductivity | $W\cdot m^{-1}\cdot K^{-1}$ |
| $t^{shell}$ | Shell thickness | m |
| $u_0$ | Superficial gas velocity | $m\cdot s^{-1}$ |
| $y_i$ | Molar fraction of species $i$ | |
| $h$ | Convective thermal coefficient | $W\cdot m^{-2}\cdot K^{-1}$ |
| $A$ | Cross section area | $m^2$ |
| $D$ | Diameter | m |
| $L$ | Reactor length | m |
| $P$ | Total pressure | Pa |
| $T$ | Temperature | K |
| $U$ | Global Coefficient of heat transfer | $W\cdot m^{-2}\cdot K^{-1}$ |
| $r$ | Reactor radius | m |
| $v$ | Atomic diffusion volume | $cm^3\cdot mol^{-1}$ |
| $z$ | Dimensionless axial coordinate | Dimensionless |
| $\lambda_{ea}$ | Heat axial dispersion coefficient | $W\cdot m^{-1}\cdot K^{-1}$ |
| $\lambda$ | Thermal conductivity | $W\cdot m^{-1}\cdot K^{-1}$ |
| $\Phi_{i,j}$ | Binary term | |
| $\mu$ | Viscosity | $Pa\cdot s$ |
| $\rho$ | Density | $kg\cdot m^{-3}$ |

## Physical Properties

To solve the kinetic model the mass axial dispersion coefficient $D_{ea}$, the heat axial dispersion coefficient $\lambda_{ea}$, the specific heat capacity $C_{p,f}$, the fluid viscosity $\mu_f$ and the global coefficient of heat transfer $U$ need to be obtained. These properties were calculated for 200 equidistant points along the reactor length as a function of the mixture composition, temperature, and pressure. Furthermore, as seen in the membrane reactor model (Section 2.1), the properties were calculated both for the retentate and the permeate zones of the membrane reactor.

To calculate $D_{ea}$, $\lambda_{ea}$, $C_{p,f}$ and $\mu_f$, it was firstly necessary to consider the thermodynamic properties of all species involved, which are presented in Table S1. The properties considered in the table are the molecular weight $M_i$, the critical temperature $T_{c,i}$, and the critical pressure $P_{c,i}$.

**Table S1.** Thermodynamic properties for the species considered [1].

| | $M_i$ / $g \cdot mol^{-1}$ | $T_{c,i}$ / K | $P_{c,i}$ / bar |
|---|---|---|---|
| $CO_2$ | 44.01 | 304.2 | 73.88 |
| $H_2$ | 2.02 | 33.19 | 13.13 |
| $H_2O$ | 18.02 | 647.1 | 220.55 |
| $CH_4$ | 16.04 | 190.7 | 46.00 |
| $N_2$ | 28.01 | 126.2 | 34.00 |

Employing the thermodynamic properties presented the following dimensionless numbers (Reynolds, Prandtl and Schmidt) were calculated for the fluid mixture according to Equations S1, S2 and S3 [1].

$$\mathrm{Re} = \frac{\rho_f \times u_0 \times d_p}{\mu_f} \tag{S1}$$

$$Pr = \frac{\mu_f \times C_{p,f}}{\lambda_f} \tag{S2}$$

$$Sc = \frac{\mu_f}{\rho_f \times D_m} \tag{S3}$$

The thermal conductivity of the fluid mixture is represented by $\lambda_f$ and $D_m$ stands for the molecular diffusion of the fluid mixture, estimated using Equation S4 [2].

$$D_m = \sum_{i=1}^{5} D_{m,i} \times y_i \tag{S4}$$

In the previous equation, $D_{m,i}$ is the molecular diffusion of the species $i$, determined by Equation S5 [2].

$$D_{m,i} = \frac{1 - y_i}{\sum_{j=1}^{5} \frac{y_j}{D_{i,j}}} \tag{S5}$$

In this equation, $D_{i,j}$ represents the binary diffusivity for the species $i$ and $j$, and is calculated by the Fuller method (Equation S6) with $T$ in kelvin, $P$ in atmospheres and $M$i in g·mol$^{-1}$ [1].

$$D_{i,j} = \frac{10^{-3} \times T^{1.75}\left(\frac{1}{M_i} + \frac{1}{M_j}\right)}{P \cdot \left(\left(\sum v\right)_i^{1/3} + \left(\sum v\right)_j^{1/3}\right)^2} \tag{S6}$$

The sum of the atomic diffusion volumes for species $i$ and $j$ is represented by $(\sum v)_i$ and $(\sum v)_j$ respectively. The values considered are presented in Table S2.

**Table S2.** Sum of the atomic diffusion volumes for the compounds considered [1].

| | $\sum v$ / $cm^3 \cdot mol^{-1}$ |
|---|---|
| **$CO_2$** | 26.9 |
| **$H_2$** | 7.07 |
| **$H_2O$** | 12.7 |
| **$CH_4$** | 24.4 |
| **$N_2$** | 17.9 |

The fluid mixture density was calculated according to Equation S7. In addition, the specific heat capacity of the gas mixture was calculated by Equation S8 [1], with $M_i$ in kg·mol$^{-1}$.

$$\rho_f = \frac{P}{R \times T} \sum_{i=1}^{5} (y_i \times M_i) \tag{S7}$$

$$C_{p,f} = \sum_{i=1}^{5} \frac{y_i \times C_{p,i}}{M_i} \tag{S8}$$

$C_{p,i}$ is the molar heat capacity of species $i$, calculated by Equation S9 [1].

$$C_{p,i} = A + B \times T + C \times T^2 + D \times T^{-2} \tag{S9}$$

The coefficients $A$, $B$, $C$ and $D$ for each species are shown in Table S3.

**Table S3.** Coefficients used to estimate $C_{p,i}$.

| | $A$ / $J \cdot mol^{-1} \cdot K^{-1}$ | $B \times 10^3$ / $J \cdot mol^{-1} \cdot K^{-2}$ | $C \times 10^6$ / $J \cdot mol^{-1} \cdot K^{-3}$ | $D \times 10^{-5}$ / $J \cdot mol^{-1} \cdot K$ |
|---|---|---|---|---|
| **$CO_2$** | 5.457 | 1.045 | 0.000 | −1.157 |
| **$H_2$** | 3.249 | 0.422 | 0.000 | 0.083 |
| **$H_2O$** | 3.470 | 1.450 | 0.000 | 0.121 |
| **$CH_4$** | 1.702 | 9.081 | −2.164 | 0.000 |
| **$N_2$** | 3.280 | 0.593 | 0.000 | 0.040 |

The fluid mixture viscosity is calculated employing the Wilke method (Equation S10) [3]. Additionally, the binary term $\phi_{i,j}$ is calculated by Equation S11 [3].

$$\mu_f = \sum_{i=1}^{5} \frac{y_i \times \mu_i}{\sum_{j=1}^{5} (y_i \times \phi_{i,j})} \tag{S10}$$

$$\phi_{i,j} = \frac{\left[1 + \sqrt{\frac{\mu_i}{\mu_j} \times \left(\frac{M_j}{M_i}\right)}\right]^2}{\left[8 \times \left(1 + \frac{M_i}{M_j}\right)\right]^{1/2}} \tag{S11}$$

The viscosity of species $i$ can be calculated by Equation S12 [1].

$$\mu_i = \frac{A \times T^B}{1 + \frac{C}{T} + \frac{D}{T^2}} \tag{S12}$$

The coefficients $A$, $B$, $C$ and $D$ used to estimate the viscosities are presented in Table S4.

**Table S4.** Coefficients to calculate the viscosity of the compounds considered.

| | *A* | *B* | *C* | *D* |
|---|---|---|---|---|
| $\mathbf{CO_2}$ | $2.15 \times 10^{-6}$ | 0.46 | 290.00 | 0.00 |
| $\mathbf{H_2}$ | $1.80 \times 10^{-7}$ | 0.69 | −0.59 | 140.00 |
| $\mathbf{H_2O}$ | $1.71 \times 10^{-8}$ | 1.11 | 0.00 | 0.00 |
| $\mathbf{CH_4}$ | $5.25 \times 10^{-7}$ | 0.59 | 105.67 | 0.00 |
| $\mathbf{N_2}$ | $6.56 \times 10^{-7}$ | 0.61 | 54.71 | 0.00 |

The thermal conductivity of the fluid is obtained by the Wassijewa method (Equation S13). Furthermore, the thermal conductivity of the compound $i$ is obtained by Eucken's equation (Equation S14) [3].

$$\lambda_f = \sum_{i=1}^{5} \frac{y_i \times \lambda_i}{\sum_{j=1}^{5}(y_i \times A_{i,j})} \tag{S13}$$

$$\lambda_i = \mu_i \times \left(1.25 \times \frac{\mathrm{R}}{M_i} + \frac{C_{p,i}}{M_i}\right) \tag{S14}$$

The binary interaction parameter was calculated by Equation S15 [3].

$$\mathrm{A_{i,j}} = \frac{\left[1 + \left(\frac{\lambda_{\mathrm{tr,i}}}{\lambda_{\mathrm{tr,j}}}\right)^{1/2} + \left(\frac{\mathrm{M_i}}{\mathrm{M_j}}\right)^{1/4}\right]^2}{\left[8 \times \left(1 + \frac{\mathrm{M_i}}{\mathrm{M_j}}\right)\right]^{0.5}} \tag{S15}$$

The ratio between the translational thermal conductivities, $\frac{\lambda_{tr,i}}{\lambda_{tr,j}}$, can be obtained employing Equation S16 [3].

$$\frac{\lambda_{tr,i}}{\lambda_{tr,j}} = \frac{\Gamma_j\left[\exp\left(0.0464 \times T_{r,i}\right) - \exp\left(0.2412 \times T_{r,i}\right)\right]}{\Gamma_i\left[\exp\left(0.0464 \times T_{r,j}\right) - \exp\left(0.2412 \times T_{r,j}\right)\right]} \tag{S16}$$

$\lambda_{tr,i}$ stands for the reduced temperature of the species $i$ and is equal to $T/T_{c,i}$. Furthermore, $\Gamma_i$ ($\mathrm{m{\cdot}K{\cdot}W^{-1}}$) is obtained by Equation S17 [3] with $M_i$ in $\mathrm{kg{\cdot}mol^{-1}}$.

$$\Gamma_i = 210\left(\frac{T_{c,i} \times {M_i}^3}{{P_{c,i}}^4}\right)^{1/6} \tag{S17}$$

The mass axial dispersion is obtained through Equation S18 [1].

$$\mathrm{D_{ea}} = \frac{\mathrm{u_0} \times \mathrm{d_p}}{\varepsilon_\mathrm{b} \times \mathrm{Pe}} \tag{S18}$$

The Peclet number ($Pe$) is a function of the Reynolds ($Re$) and Schmidt ($Sc$) dimensionless numbers (Equation S19) [1]

$$\frac{1}{\mathrm{Pe}} = \frac{0.3 \times \varepsilon_\mathrm{b}}{\mathrm{Re} \times \mathrm{Sc}} + \frac{0.5}{1 + \frac{3.8}{\mathrm{Re} \times \mathrm{Sc}}} \tag{S19}$$

Finally, the axial heat dispersion coefficient is obtained through the Wakao and Funazkri correlation (Equation S20).

$$\lambda_\mathrm{ae} = 7\lambda_\mathrm{f} + 0.5 \times \rho_\mathrm{f} \times \mathrm{u_0} \times \mathrm{d_p} \times \mathrm{C_{p,f}} \tag{S20}$$

For the traditional reactor, the global coefficient of heat transfer ($U^R$) is obtained by Equation S21.

$$\frac{1}{\mathrm{U^R}} = \frac{1}{\mathrm{h^R}} + \frac{\mathrm{A^R} \times \ln\left(\left(\mathrm{r^R} + \mathrm{t^{shell}}\right)/\mathrm{r^R}\right)}{2 \times \pi \times \mathrm{L} \times \mathrm{k^{shell}}} + \frac{\mathrm{A^R}}{\mathrm{A^{shell}}} \times \frac{1}{\mathrm{h^{out}}} \tag{S21}$$

$t^{shell}$ is the shell thickness; $k^{shell}$ is the shell thermal conductivity (equal to 14.4 W·m$^{-1}$·K$^{-1}$, for stainless steel) and $h^{out}$ is the convective heat coefficient in the oven (equal to 50 W·m$^{-3}$·K$^{-1}$, natural convection of air) [1].

For the membrane reactor, the global coefficient of heat transfer from the retentate to the permeate side ($U^R$) is determined by Equation S22.

$$\frac{1}{U^R} = \frac{1}{h^R} + \frac{A^R \times \ln\left((r^R + t^m)/r^R\right)}{2 \times \pi \times L \times k^m} \quad \text{(S22)}$$

$t^m$ is the membrane thickness and $k^m$ is the membrane conductivity (equal to 71.2 W·m$^{-1}$·K$^{-1}$, for palladium).

Additionally, the heat transfer coefficient between the gaseous phase and the membrane ($h^R$) is calculated by Equation S23.

$$h^R = 0.17 \times \frac{\lambda_f}{d_p} \times \left(\frac{Pr}{0.7}\right)^{1/3} \times Re^{0.79} \quad \text{(S23)}$$

The global coefficient of heat transfer ($U^P$) from the permeate side to the exterior is estimated by Equation S24.

$$\frac{1}{U^P} = \frac{1}{h^P} + \frac{{A_L}^P \times \ln\left(r^{shell}/r^P\right)}{2 \times \pi \times L \times k^{shell}} + \frac{{A_L}^P}{{A_L}^{shell}} \times \frac{1}{h^{out}} \quad \text{(S24)}$$

The coefficient $h^P$ was estimated according to Equations S25, S26 and S27 [1]:

$$h^P = \frac{{\lambda_f}^P}{D_h} \times (Nu)_{annular} \quad \text{(S25)}$$

$$(Nu)_{annular} = 0.86 \times (Nu)_{cylinder} \left(\frac{r^P}{r^R + t^m}\right)^{0.16} \quad \text{(S26)}$$

$$(Nu)_{cylinder} = 3.66 + \frac{0.0668 \times \left(\frac{2 \times r^P}{L}\right) \times Re \times Pr}{1 + 0.04\left[\left(\frac{2 \times r^P}{L}\right) \times Re \times Pr\right]^{2/3}} \quad \text{(S27)}$$

in which ${\lambda_f}^P$ stands for the gas thermal conductivity in the permeate side, $D_h$ is the hydraulic diameter, $(Nu)_{annular}$ is the Nusselt dimensionless number for the flow in noncircular ducts and $(Nu)_{cylinder}$ is the Nusselt dimensionless number for flow in round cylinders.

## Simulation Results

*1. Effect of the temperature in the MR*

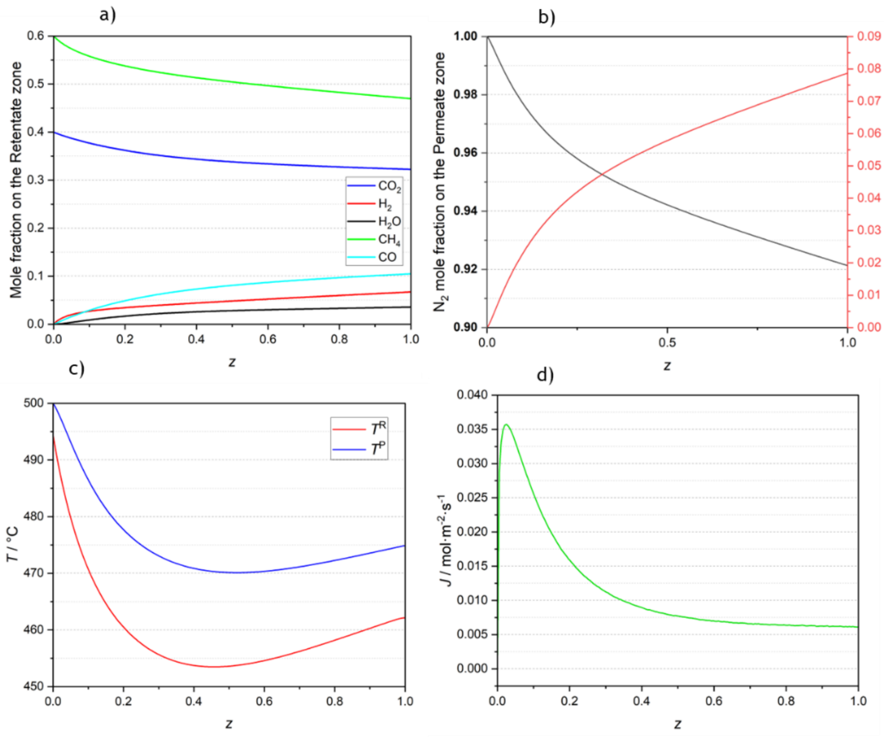


**Figure S1.** Mole fractions on the retentate (**a**) and permeate (**b**) zones, temperature (**c**), and permeated molar flux of hydrogen (**d**) along the reactor, operating with a feed temperature of 500 °C.

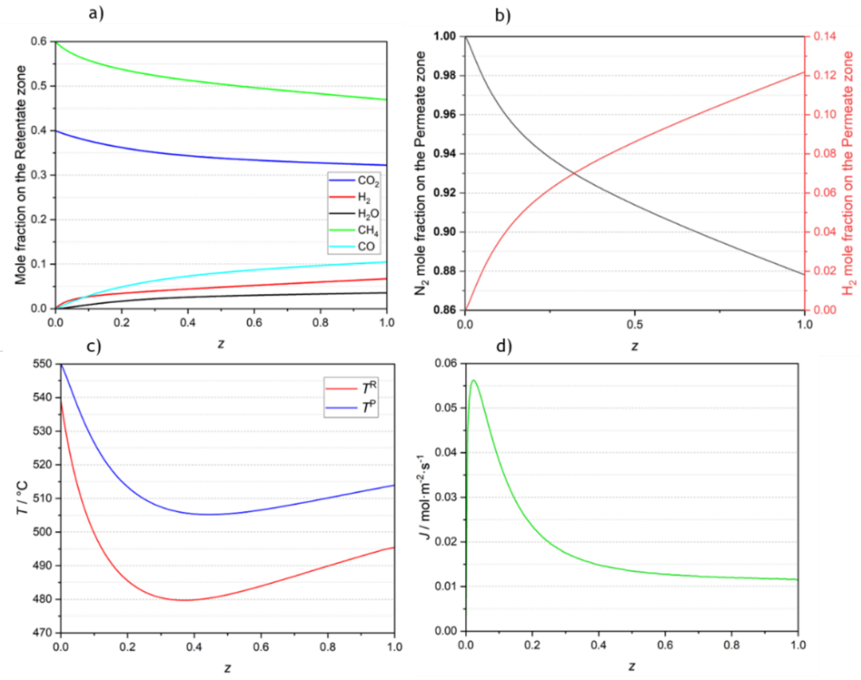


**Figure S2.** Mole fractions on the retentate (**a**) and permeate (**b**) zones, temperature (**c**), and permeated molar flux of hydrogen (**d**) along the reactor, operating with a feed temperature of 550 °C.

## 2. *Effect of the pressure in the MR*

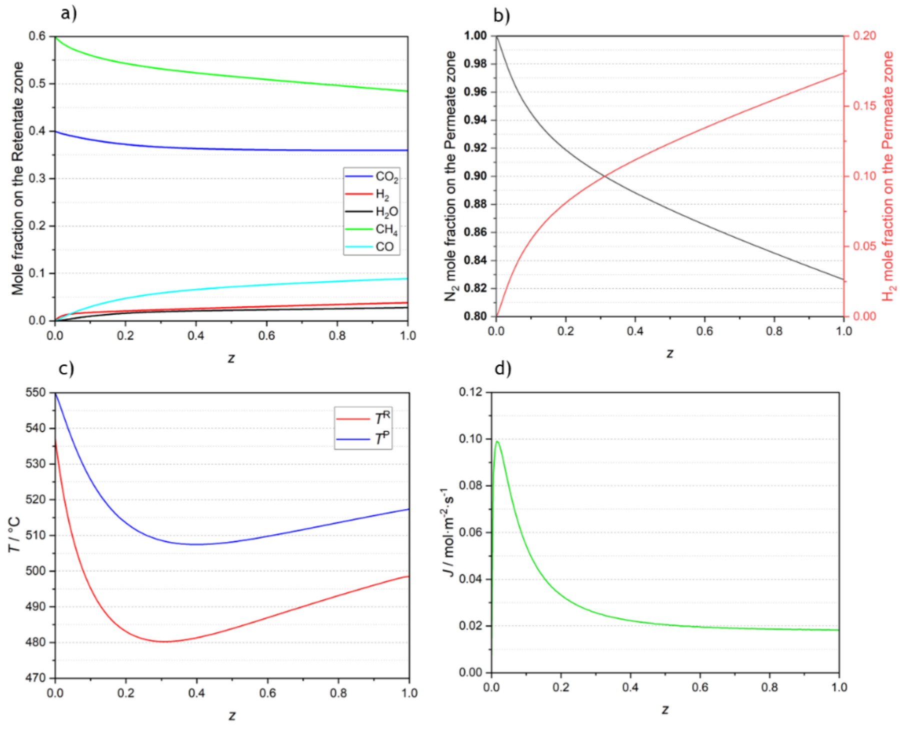


**Figure S3.** Mole fractions on the retentate (**a**) and permeate (**b**) zones, temperature (**c**), and permeated molar flux of hydrogen (**d**) along the reactor, operating with a feed pressure of 5 bar.

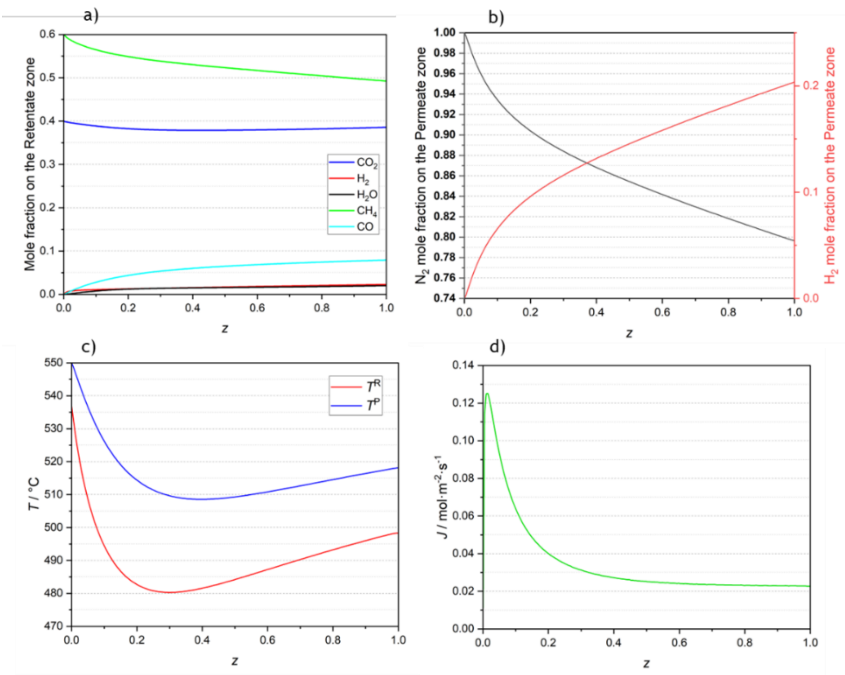


**Figure S4.** Mole fractions on the retentate (**a**) and permeate (**b**) zones, temperature (**c**), and permeated molar flux of hydrogen (**d**) along the reactor, operating with a feed pressure of 10 bar.

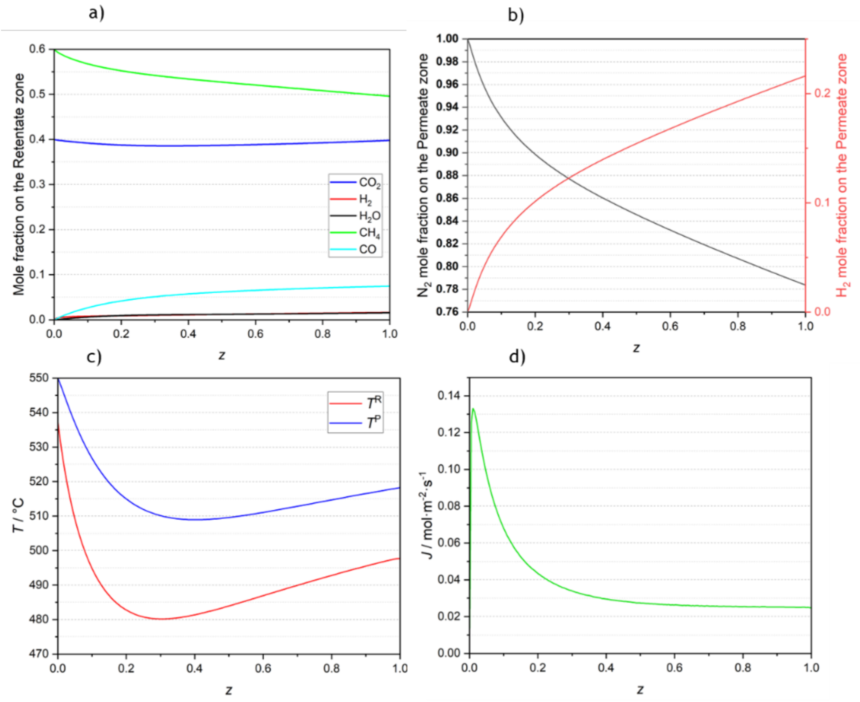


**Figure S5.** Mole fractions on the retentate (**a**) and permeate (**b**) zones, temperature (**c**), and permeated molar flux of hydrogen (**d**) along the reactor, operating with a feed pressure of 15 bar.

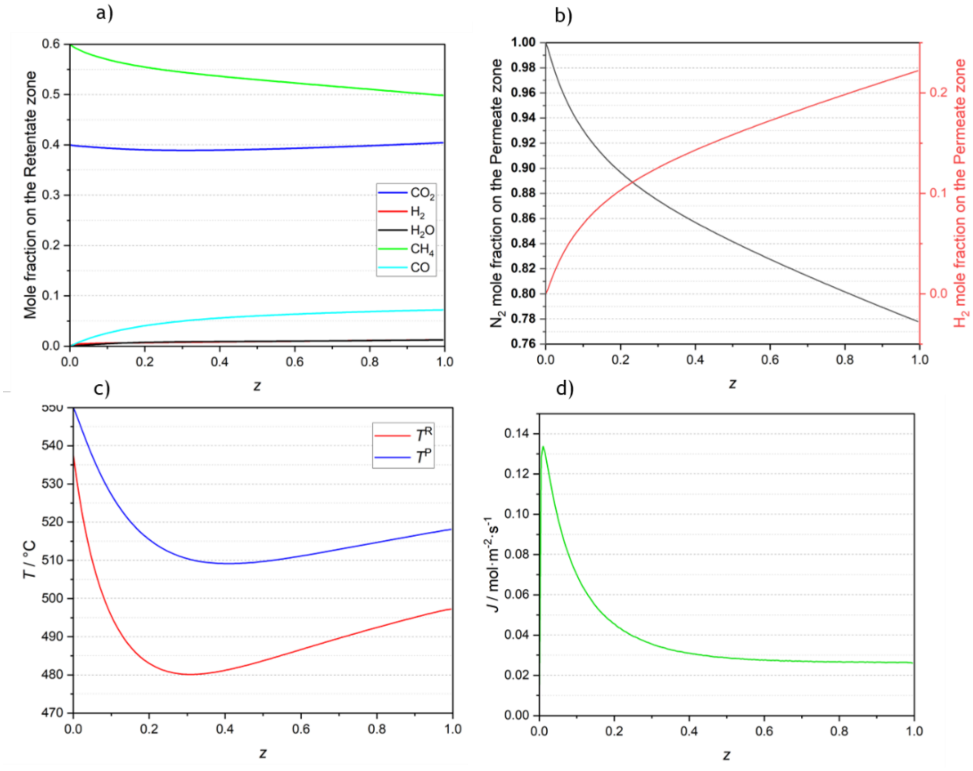


**Figure S6.** Mole fractions on the retentate (**a**) and permeate (**b**) zones, temperature (**c**), and permeated molar flux of hydrogen (**d**) along the reactor, operating with a feed pressure of 20 bar.

*3. Effect of biogas composition in the MR*

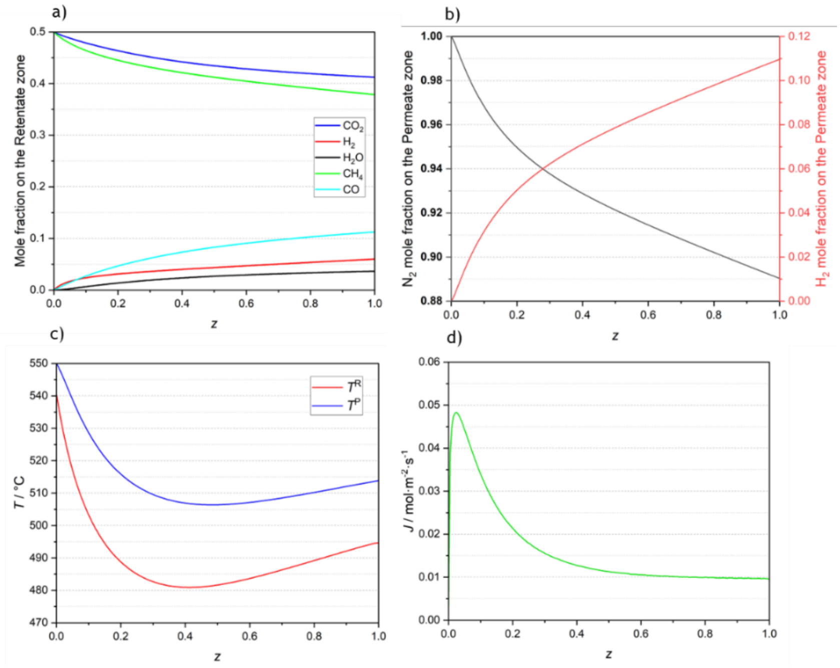


**Figure S7.** Mole fractions on the retentate (**a**) and permeate (**b**) zones, temperature (**c**), and permeated molar flux of hydrogen (**d**) along the reactor, operating with a 1/1 $CH_4/CO_2$ inlet molar ratio and a GHSV of 41.0 $L_{STP}·h^{-1}·gcat^{-1}$.

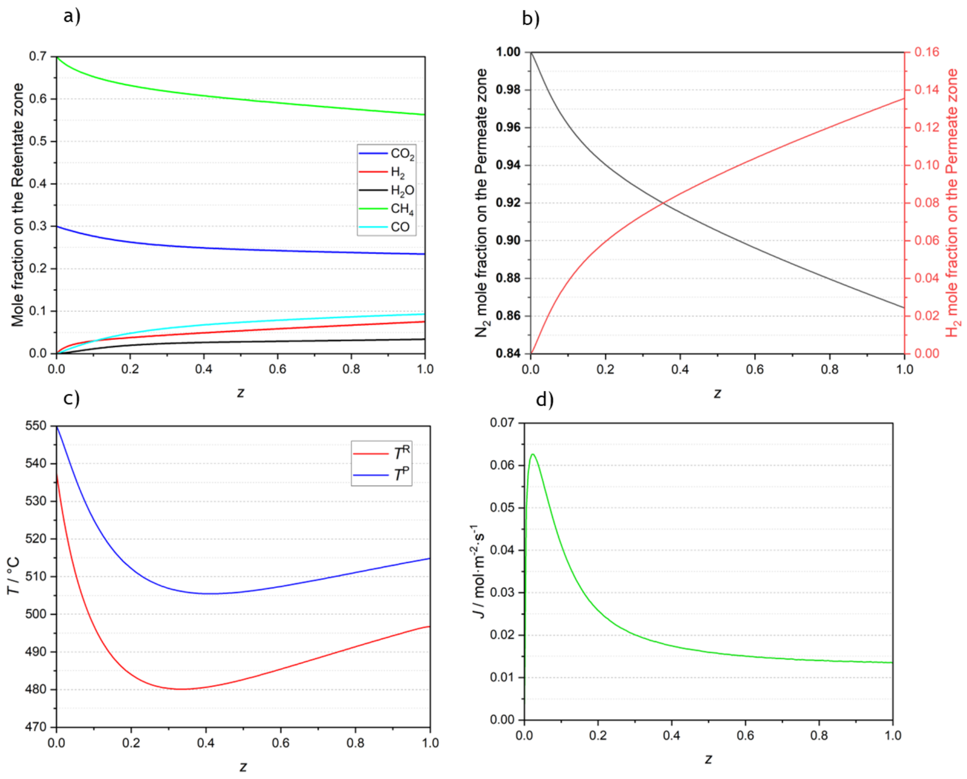


**Figure S8.** Mole fractions on the retentate (**a**) and permeate (**b**) zones, temperature (**c**), and permeated molar flux of hydrogen (**d**) along the reactor, operating with a 7/3 $CH_4/CO_2$ inlet molar ratio and a GHSV of 30.0 $L_{STP}·h^{-1}·gcat^{-1}$.

*4. Comparison between MR and TR*

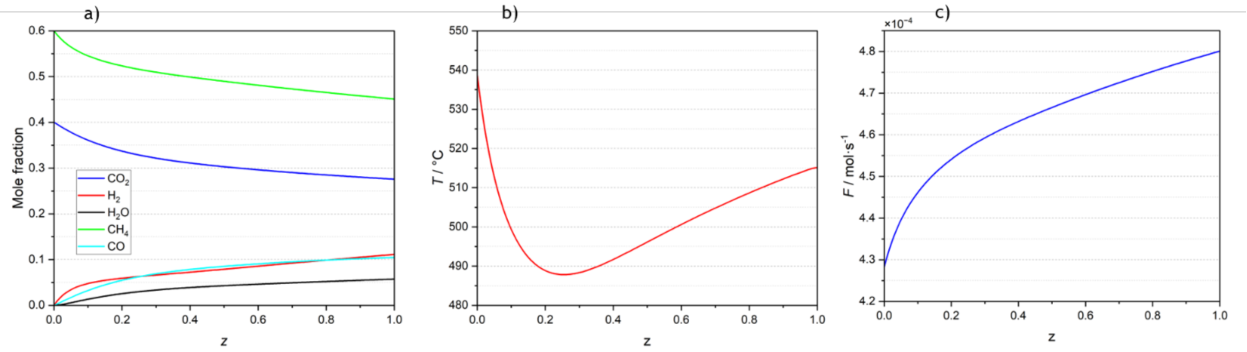


**Figure S9.** Mole fractions (**a**), temperature (**b**), and molar flowrate (**c**) along the traditional reactor length, operating with a feed temperature of 550 °C, a feed pressure of 2 bar and a 3/2 $CH_4/CO_2$ inlet molar ratio.

## References


1. Perry, R. H., D.W. Green (1999). Perry's Chemical Engineers' Handbook. McGraw-Hill Education, New York.
2. Rodrigues, A.E.; Madeira, L.M.; Wu, Y.-J.; Faria, R. (2017). Sorption Enhanced Reaction Processes. World Scientific Publishing Europe Ltd, New Jersey.
3. Poling, B. E., Prausnitz, J. M., & O'Connell, J. P. (2001). Properties of Gases and Liquids (Fifth edition. ed.). McGraw-Hill Companies, New York.
